# A Pre-Hispanic Head

**DOI:** 10.1371/journal.pone.0002053

**Published:** 2008-04-30

**Authors:** Raffaella Bianucci, Maria Jeziorska, Rudy Lallo, Grazia Mattutino, Massimo Massimelli, Genevieve Phillips, Otto Appenzeller

**Affiliations:** 1 Laboratory of Anthropology, Department of Animal and Human Biology, University of Turin, Turin, Italy; 2 Division of Regenerative Medicine, University of Manchester, Manchester, United Kingdom; 3 Fondazione Pinna Pintor and Studio Futura, Turin, Italy; 4 Laboratory of Criminalistic Sciences, Department of Anatomy, Pharmacology and Legal Medicine, University of Turin, Turin, Italy; 5 Legal Medicine Office, Turin, Italy; 6 Fluorescence Microscopy Facility, Cancer Research and Treatment Center, University of New Mexico Albuquerque, New Mexico, United States of America; 7 New Mexico Health Enhancement and Marathon Clinics Research Foundation, Albuquerque, New Mexico, United States of America; Quinnipiac University, United States of America

## Abstract

This report on a male head revealed biologic rhythms, as gleaned from hydrogen isotope ratios in hair, consistent with a South-American origin and Atomic Mass Spectrometry radiocarbon dating (AMS) compatible with the last pre-Hispanic period (1418–1491 AD, 95.4% probability). Biopsies showed exceptionally well-preserved tissues. The hair contained high levels of toxic elements (lead, arsenic and mercury) incompatible with life. There was no evidence for lead deposition in bone consistent with *post-mortem* accumulation of this toxic element in the hair. We propose that the high content of metals in hair was the result of metabolic activity of bacteria leading to metal complexation in extra cellular polymeric substances (EPS). This is a recognized protective mechanism for bacteria that thrive in toxic environments. This mechanism may account for the tissues preservation and gives a hint at soil composition where the head was presumably buried. Our results have implications for forensic toxicology which has, hitherto, relied on hair analyses as one means to reconstruct *pre-mortem* metabolism and for detecting toxic elements accumulated during life. Our finding also has implications for other archaeological specimens where similar circumstances may distort the results of toxicological studies.

## Introduction

**Figure 1:**
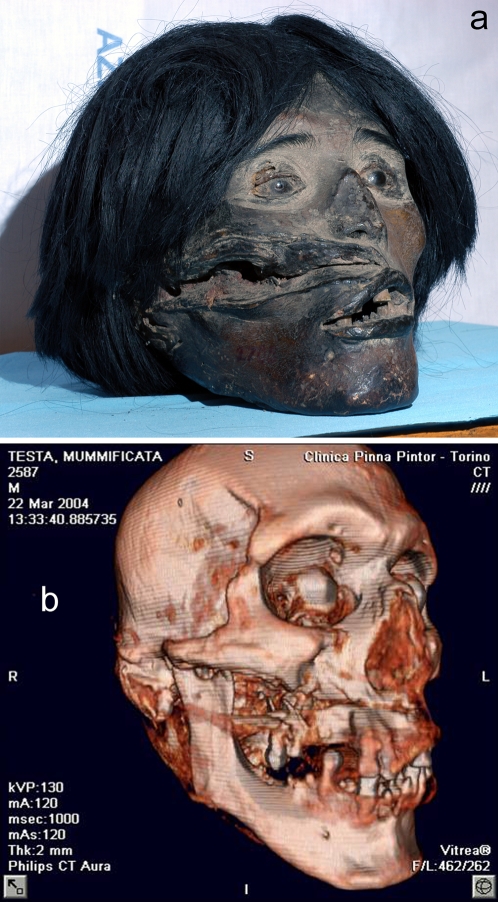
A South-American head now housed in Turin's Museum of Anthropology and Ethnography: (a) The specimen (TSA n° 1); (b) 3D virtual reconstruction of the head showing the fracture of the right maxilla and of the right mandibular condyle. The fractures are not communicating with the oropharinx. By inference it is unlikely that blood from the injury would have entered the trachea and caused death by ‘drowning’.

A South-American male (TSA n°1) whose head is now housed in Turin's Museum of Anthropology and Ethnography was in his twenties when he died some 600 years ago. A huge scar (14.5 cm×2.9 cm) is visible on his right cheek overlaying a fracture **(** **Fig. 1** **)**.

An earlier report on this specimen detailed the results of two series of CT scans to determine age and cause of death of this young man [**1**]. Subsequently an endoscopic examination of the interior of the cranium through the foramen magnum revealed the presence of a white-yellowish ‘waxy’ material filling the skull.

Since these initial analyses, several additional studies confirmed and amplified evidence for the extraordinary preservation of this head.

Here we present a hypothesis to explain this unusual preservation. We also amplify the evidence, previously obtained, for the preservation of the specimen using different techniques.

We posit that *post-mortem* bacterial activity in the tissues concentrated lead, arsenic and mercury from the soil. These toxic elements, subsequently, inhibited other bacterial growths which, under ordinary circumstances, are detrimental to tissue preservation. Our findings imply that the reliance of forensic toxicology on hair analyses as a reflection of *pre-mortem* metabolism and toxic exposure might, occasionally, be misguided.

## Results

Radiocarbon dating places the head into the last pre-Hispanic period [**2**], between 1418–1491 A.D. (95% probability range). Stable isotopes were analyzed in hair. The measured δ^13^C value was −18.1‰ and the measured value of δ^15^N was +12.4 ‰.

**Figure 2:**
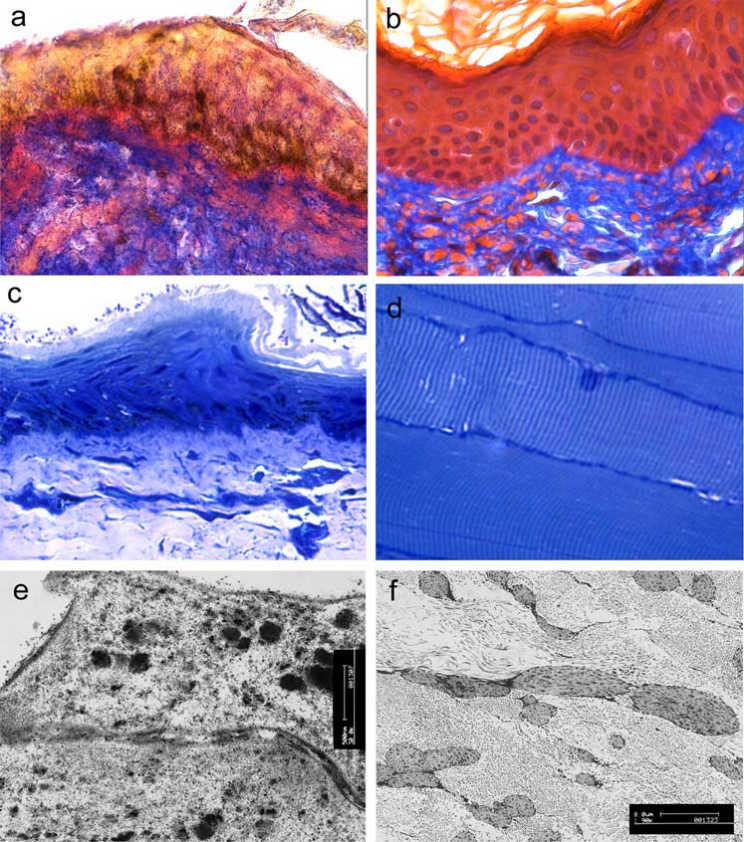
Light and transmission electron microscopy of skin and soft tissues from TSA n°1: (a) 5 µm section from the skin on the edge of facial wound stained with Masson's trichrome compared with contemporary skin (b); (c) semi-thin resin sections from the same area as (a) showing clearly visible nuclei in the epidermis. Note, bacteria and fungal hyphae on the surface; (d) biopsy from the neck region showing well-preserved muscle fibres with distinctive striations (Toluidine blue staining); (e) ultra-thin section showing desmosomes between two keratinocytes; (f) ultra-thin section showing well-preserved collagen and elastic fibres in the dermis.

Biopsies examined by light and electron microscopy revealed exceptionally well-preserved tissues. At low magnification (250×), in H & E (haematoxylin-eosin), the various components of the cutaneous samples such as epidermis, dermis, pilo-sebaceous glands and sweat glands were easily identifiable. **(** **Fig. 2** **)**

The epidermis was covered by a thick brownish layer made of an amorphous material occasionally interrupted by granulations. The epidermis showed normal structure with the different layers easily identifiable; the nuclei and the edges of cytoplasm were clearly detectable. Desmosomes were observable in the Malpighian layer as well as melanin pigment in the cytoplasm's basal layer **(** **Fig. 2** **)**.

The underlying dermis, made up of collagen and elastic fibres, was partially decomposed and showed fungal hyphae and spores identified by periodic-acid Shiff reaction (PAS) and Grocott stains. Some fungal contaminants were also present in the epidermis and in the hypodermis. There was normal immunoreactivity for cytokeratins of the epidermis and of the glandular epithelium **(** **Table 1** **)**.

**Table 1:** Comparison of immunohistochemical staining of epithelial, vascular and muscle tissues taken from TSA n°1 and from a contemporary specimen to show the preservation of immunoreactivity in the mummified skin.

	TSA n°1	Contemporary tissue
**α-SMC actin**	+	+++
**CD68**	−	+++
**elastin**	++	+++
**collagen type I**	++	+++

α-SMC actin = alpha smooth muscle cell actin; CD68 = Cluster of Differentiation 68.

**Figure 3:**
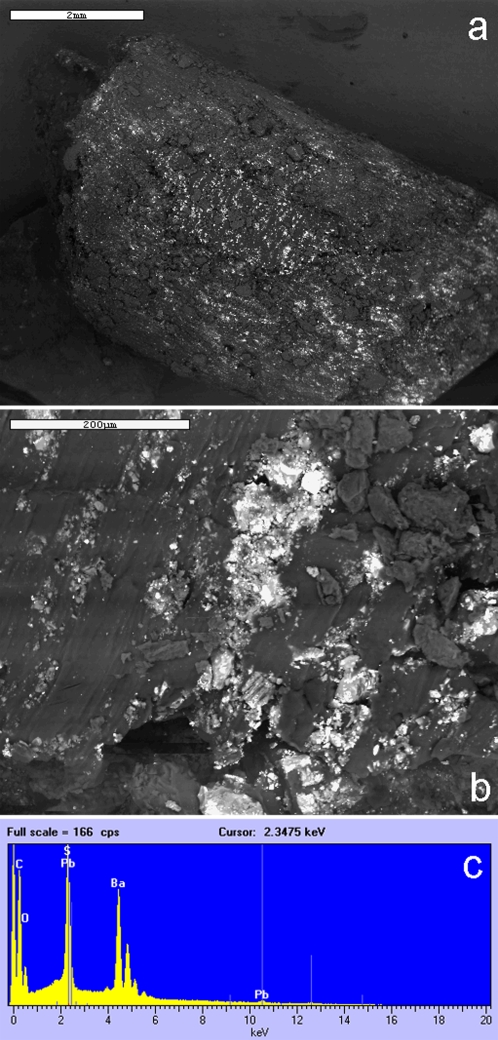
Scanning electron microscopy appearance of the ‘wax-like’ material filling the skull: (a, b) low power and higher power; (c) micro-analytical spectrum showing accumulation of barium sulphate and lead.

Microanalysis performed on the material filling the skull showed the presence of barium sulphate and lead. The distribution of these metals was not homogeneous showing accumulations in some areas and dispersal in others **(** **Fig. 3** **)**.

Confocal microscopic appearances of contemporaneous, blond and black hairs, were compared with hair from the specimen and with hair obtained from a naturally mummified South-American female mummy also belonging to Turin's Museum of Anthropology [**3**].

**Figure 4:**
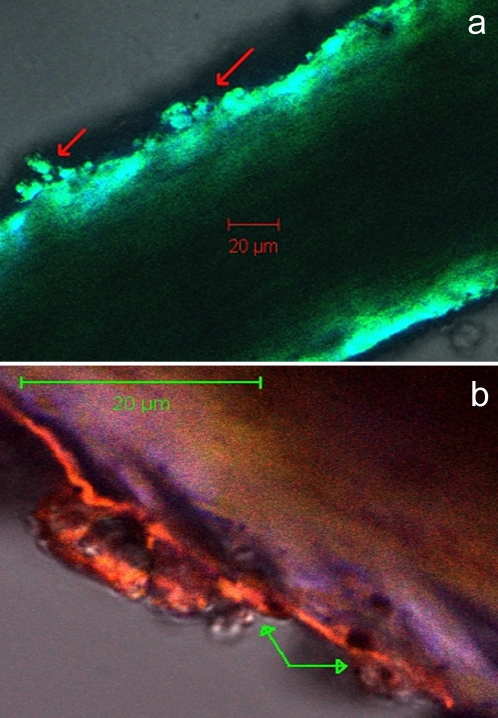
Confocal microscopic appearance of ancient scalp hair: (a) Extracellular polymeric substances (EPS) on hair surface of TSA n°1 (arrows); (b) EPS on hair surface of a naturally mummified specimen. The confocal microscopic features of these bacterial products are indistinguishable in the two specimens.

**Figure 5:**
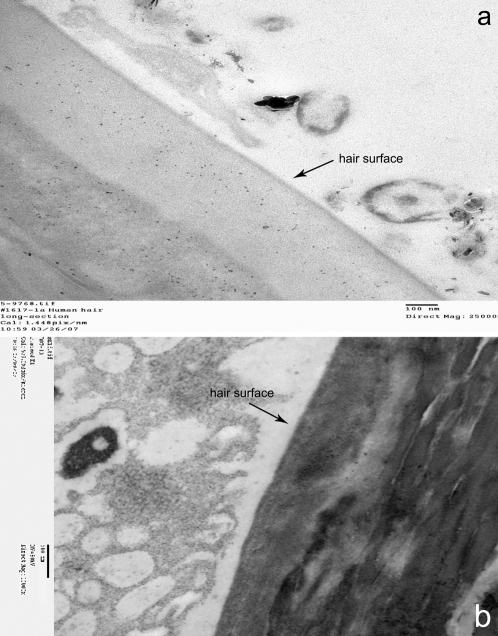
Transmission electron microscopy of ancient scalp hair: (a) EPS on TSA n°1 hair. Note bacterial and fungal profiles; (b) EPS surrounding a bacterium on the surface of another mummy's hair, supporting the bacterial origin of the amorphous material.

Mummified hair showed irregular cracks easily identifiable on cross-section, not found in contemporaneous hair. On the surface of mummified hair various shaped accretions with numerous holes of different sizes and shapes were identifiable. These accretions and hair shafts were autofluorescent. We propose that these structures represent extra-cellular polymeric substances (EPS) also known as bio-films deposited on the surface of the hair by bacterial metabolism **(** **Figs. 4** **&** **5** **)**. Electron microscopy of hair confirmed the presence of EPS in our specimen. In support of this interpretation we show an electron micrograph obtain from another mummy's hair showing EPS with one bacterium still in place **(** **Fig. 5** **)**.

We further examined the hair by Inductively Coupled Plasma/ Mass Spectrometry (ICP/MS) after the usual thorough washing and cleaning to remove surface contaminants (using 0.2% Triton-X and then rinsed several times with double distilled water).

ICP/MS showed a lead content of 8680 mcg/g (normal concentrations of lead in hair: 5–29 mcg/g), an arsenic concentration of 38 mcg/g (normal concentrations of arsenic in hair: 0.03–3 mcg/g hair) and mercury concentration of 6410 mcg/g (normal concentration of mercury 0.4–1.2 mcg/g hair) **(** **Table 2** **)**.

**Table 2:** ICP/MS elemental analysis of hair taken from TSA n°1, a naturally mummified South-American specimen and a contemporaneous sample.

Elements	TSA n°1	Naturally mummified	Contemporaneous hair
**Arsenic**	**38**	**3**	0.03–3
Calcium	3.700	8.900	NA
**Mercury**	**6410**	**<1**	0.4–1.2
Potassium	780	14.500	NA
Magnesium	520	2.300	NA
Sodium	1600	5.760	NA
**Lead**	**8680**	**200**	5–29
**Zinc**	**1600**	**360**	130–220

Note the enormous levels of lead, mercury, zinc and arsenic in our study specimen compared to the naturally mummified and contemporaneous specimens. Calcium, potassium, magnesium and sodium are, by contrast, much increased in the hair of the naturally mummified specimen.

Values are expressed in mcg/g of hair; NA = not applicable.

## Discussion

It is well accepted that life is found everywhere even in the most unlikely places such as acid fluids that can dissolve steel and at pressures and temperatures found in hospital autoclaves used to sterilize surgical instruments. These organisms are now known as “extremophiles”, they are *Archaea* that are classed in the “third domain of life”, they differ from bacteria, but like bacteria, are single celled; unlike bacteria, however, they are not known to cause disease.

*Archaea* were discovered some 30 years ago and their number keeps on increasing with an accelerating pace of new discoveries [**4**]. Bacteria have also been found to thrive in places previously thought incompatible with “normal” metabolic processes. Methanogenes, for example, get energy from combining hydrogen and carbon dioxide to produce the greenhouse gas methane thought to contribute to global warming.

The bacterium *Ralstonia mettalidurans* is resistant to metals that are highly toxic to other bacteria. It grows in the presence of millimolar concentrations of dissolved silver, cadmium, cobalt, lead, mercury nickel and chromium. This bacterium has also been found to precipitate gold from toxic gold chloride complexes and it continues to grow in AuCl_4_^−^ after selection for resistance to this toxin [**5**].

The enormous levels of toxic elements we found in the hair are incompatible with life: they could not have been incorporated by human metabolism into the hair and, by inference, must have been deposited *post-mortem*. These very high levels also exclude acute poisoning because there would not have been enough time before death for the hair to grow sufficiently to incorporate the toxic elements; human hair grows at approximately 16 cm per year.

We set out here to support our hypothesis that the high levels of toxic elements in the hair of our specimen were deposited by soil bacteria. By analogy, the mechanism for the concentration of toxic elements in the hair of our specimen is postulated to be akin to that adopted by *Ralstonia mettalidurans* concentrating gold complexes in gold mines to produce gold nuggets [**5**].

Our initial postulate that appropriate imaging of the hair would reveal bio-films secreted by the bacteria to protect themselves from the toxic surroundings was amply confirmed by confocal and electron microscopy.

Hair is renewed throughout life; it has also a defined variability in growth. Each hair follicle cycles between three stages: growth, involution and rest. Human hair remains in the growth-stage for 2–8 years to produce long hair.

Stable isotope ratios in tissues, including hair, depend on these ratios in food and water. And hair is an ideal material that can be used to study diets of ancient civilizations [**6**].

Thus stable isotope ratios along the length of hair contain a record of metabolic activity often spanning many years depending on the length of the hair [**7**]. The analysis of the hydrogen, carbon and nitrogen isotopes in our specimen support the contention that this young man lived in South America around the time of the Spanish conquest and survived on a diet consisting of mainly terrestrial food (C3 plants) with a small marine input.

Previous studies carried out on bone collagen and hair belonging to a 7–10 years old boy mummy found on Mount Aconcagua (Argentina), radiocarbon dated at 370±70 years before present (BP), showed a δ^13^C value of −10.8‰ and a δ^15^N value of +10.4 ‰ attributed to a predominantly maize based, non-marine diet [**8**].

In typical maize-based diets the δ^15^N value, in bone collagen, ranges from 9.0 to 9.9 ‰. In bone collagen samples of Inca-related Sausas, the measured δ^15^N ranged from 8 to 13‰ [**9**].

Earlier results [**8**] revealed that δ^15^N values of hair are generally lower than for bone collagen [**10**].

The δ^13^C value in our hair sample is consistent with a terrestrial diet (C3 plants) with a small but significant maize-based component (C4 plants) whereas the high δ^15^N value could reflect an additional small marine component.

To further support our contention that the concentrating power of bacteria that entered the hair *post-mortem* from the soil accounted for the high levels of metabolic toxic elements, we examined hair from a different mummy, also from South America, but buried in a different location.

In this specimen the visual appearance of bio-films was indistinguishable from that of our subject's hair but the elemental analysis showed high levels of different elements, in keeping with the proposition that the bacterial fauna in this burial site and the soil's elemental content determined the results of the heavy metal concentration in hair **(** **Table 2** **)**.

Our hypothesis that the high levels of heavy metals in our subject's hair were deposited by bacterial concentrating activity in bio-films has therefore some support. These toxic elements must have been deposited on the hair post-mortem by mechanism resembling those used by the bacterium *Ralstonia mettalidurans* to produce gold nuggets in some Australian gold mines [**5**].

Thus one explanation for the well-preserved tissues in our specimen is that extraordinary circumstances, bacterial growth and soil content of heavy metals conspired to preserve the tissues. Subsequently, this lead to inhibition of putrefaction normally induced by other bacteria and thus contributed to the exquisite tissue preservation.

The heavy metals, present in such abundance in the hair of our specimen, are often found in rocks associated with sulphide minerals such as pyrite. Mining exposes these rocks to the weather and rain leaches these metals due to the action of sulphuric acid produced from the sulphides in the rock. Eventually these toxic substances find their way into surface waters where the acid releases more metals from exposed rocks. This process, known as acid mine drainage (AMD) is the most persistent form of water and soil pollution caused by mining [**11**].

South-America, especially Peru and Chile, have numerous mines that have been exploited for centuries. Gold, copper, lead, zinc, manganese, antimony, bismuth, molybdenum, tin and mercury mines have all contributed to AMD and consequent water and soil pollution. Thus it is not inconceivable that soil containing large amounts of heavy metals should be widely distributed in South-America.

For bacteria to survive in such toxic surroundings they must evolve mechanisms to protect their metabolism. One such mechanism is concentrating the toxic elements in their bio-films or extra cellular polymeric substances (EPS) where they can do no harm. This mechanism of survival has been found for *Ralstonia mettalidurans* and for numerous other bacteria that thrive in highly toxic sludges concentrating for example lead and cadmium.

EPS from pure bacterial cultures are composed of proteins and low quantities of polysaccharides and uronic acid [**12**]. But those extracted from the same bacteria living in activated sludges, for example, contain high levels of cadmium, lead and nickel [**13**].

The concentration of these elements varies with the complexation power of EPS [**14**], [**15**]. Bacteria have also been used for the clean-up of toxic refuse and contaminated soils where their activity removes the toxic elements and stores the offending substances in their EPS where they can do no harm [**13**].

The extraordinary tissue preservation we found in our specimen is also consistent with a complementary explanation, however. The presence of a ‘wax-like’ substance filling the skull indicates that anthropogenic treatment of the head took place sometime after death. This substance, whose composition has not been ascertained, was poured into the skull and was also rubbed onto the skin and possibly hair for perhaps the specific purpose of preserving the tissues. Toxic elements of the kind present in the scalp hair (mainly lead) were identified, by micro-analysis, also in the deepest levels of the solidified ‘wax-like’ material within the skull.

The combined actions of the embalming substances and of soil bacteria, which concentrated the toxic elements into their EPS, may account for the superb quality of mummification of the head.

We conclude that, under certain circumstances, bacterial activity through the complexation of toxic elements in EPS may preserve tissues and we present supporting evidence for this conclusion.

Our results have implications for forensic investigations. Hair is often subjected to careful analyses after death to exclude criminal intent. But consideration of bacterial activity as a cause of accumulation of toxic elements in the tissues has not, hitherto, been considered important. Our findings suggest that bacterial activity rather than metabolic concentration of toxic elements in hair may result in the accumulation of significant amounts of toxic elements and thus may, occasionally, mislead criminal investigators.

## Materials and Methods

Biopsies were taken from the scalp, the submandibular area and at the wound's edge. Skin and muscle samples were used for immunohistochemical analyses and examined by light and electron microscopy. Immunohistochemistry was used to check the preservation of tissue antigenicity.

Samples were fixed in neutral buffered formalin, embedded in paraffin, and processed by hand. Biopsies were examined in haematoxylin-eosin (H& E) stained sections. In addition to H & E, other histochemical methods such as periodic-acid Shiff reaction (PAS), Grocott (silver methenamine), elastic fiber stain and MSB trichrome were also used. Immunoperoxidase stains were performed on de-waxed paraffin sections using an avidin-biotin horseradish peroxidase technique. Antibodies identifying epithelia, nerves, muscles and blood vessels were applied to the tissue sections after suitable antigen retrieval methods [**16]**, [17****] **(** **Table 1** **&** **Fig. 2** **)**.

Hair and skin samples taken from the occipital area were used for Atomic Mass Spectrometry radiocarbon dating [**18]**, [19], [20****].

Samples of the ‘waxy’ material filling the skull were analysed using a LEO 1430 VP scanning electron microscope (LEO Electron Microscopy Ldt, Cambridge, UK) with a Link ISIS 300 dispersive X-ray analyzer (EDX) equipped with the Cameo™ program for X-ray colour imaging (Oxford Instruments, High Wycombe, UK) [**21**], directly in variable pressure.

Elemental content of hair was determined by Inductively Coupled Plasma Mass Spectrometry (ICP/MS) and hair samples were observed by confocal microscopy using a Zeiss LSM510 META confocal microscope.
